# Adherence to Dietary and Physical Activity Guidelines in Australian Undergraduate Biomedical Students and Associations with Body Composition and Metabolic Health: A Cross-Sectional Study

**DOI:** 10.3390/nu13103500

**Published:** 2021-10-03

**Authors:** Linda A. Gallo, Tania F. Gallo, Sophia L. Young, Amelia K. Fotheringham, Johanna L. Barclay, Jacqueline L. Walker, Karen M. Moritz, Lisa K. Akison

**Affiliations:** 1 School of Biomedical Sciences, The University of Queensland, St Lucia, QLD 4072, Australia; sophia.young@uq.net.au (S.L.Y.); k.moritz1@uq.edu.au (K.M.M.); l.akison@uq.edu.au (L.K.A.); 2 North Melbourne Football Club, Arden Street, North Melbourne, VIC 3051, Australia; tania.gallo@nmfc.com.au; 3 Mater Research Institute—The University of Queensland, Translational Research Institute, Woolloongabba, QLD 4102, Australia; amelia.fotheringham@mater.uq.edu.au (A.K.F.); j.barclay@victorchang.edu.au (J.L.B.); 4 School of Human Movement and Nutrition Sciences, Faculty of Health and Behavioural Sciences, The University of Queensland, St Lucia, QLD 4072, Australia; j.walker3@uq.edu.au

**Keywords:** blood glucose, diet, exercise, insulin resistance, obesity, young adults

## Abstract

There is a paucity of data on whether Australian university students are meeting specific nutrient guidelines, and the relationship between diet and physical activity patterns with body composition and metabolic health. In this study, biomedical students from The University of Queensland were recruited (150 males and 211 females, 19–25 years), and nutritional intake (ASA24-Australia) and physical activity levels (Active Australia Survey) quantified. Body composition (height, waist circumference, body mass, BMI, and percentage body fat; BOD POD) and metabolic health (oral glucose tolerance test) were also measured. Median daily energy intake was 6760 kJ in females and 10,338 kJ in males, with more than 30% of total energy coming from energy-dense, nutrient-poor foods. Only 1 in 10 students met fruit or vegetable recommendations, with less than one third meeting recommendations for fibre, calcium, and potassium. Intakes of calcium and iron were particularly low among female students, with only 16% and 6% of students meeting the recommended dietary intake (RDI), respectively. The majority of males and almost half of all females exceeded the suggested dietary target (SDT) for sodium. Sufficient physical activity (≥150 min over ≥5 sessions per week) was met by more than 80% of students. Body composition and blood glucose concentrations were largely normal but an early sign of insulin resistance (HOMA-IR > 2.0), measured in a subset of students, was present in 21% of males and 17% of females. Modest reductions in blood glucose levels and percentage body fat were associated with increasing vigorous activity. Low intakes of fibre, calcium, and potassium could be corrected by increasing fruit, vegetable, and dairy intake, and, among females, health promotion messages focusing on iron-rich foods should be prioritised. While these nutrient deficiencies did not translate into immediate metabolic heath concerns, dietary behaviours can track into adulthood and have lasting effects on overall health.

## 1. Introduction

University students represent a young adult population with greater food choice autonomy, exposure to new food cultures, and low budgets. Given that diet is a major determinant of noncommunicable disease risk, such as obesity and diabetes mellitus [1], this is a pertinent stage of life to optimise health habits. 

Numerous international studies have reported high intake of confectionary, processed meat, and fast foods, and low consumption of cereals, legumes, nuts, fruits, and vegetables, among university students [2,3,4]. Similarly, in Australia, studies have reported that the vast majority of university students do not meet recommendations for fruit and vegetable intake [5,6], a likely consequence of energy-dense, nutrient-poor (EDNP) food intake [7]. There are no published data on whether Australian university students are meeting specific nutrient requirements, according to the Nutrient Reference Values for Australia and New Zealand [8]. However, national data in young adults aged 19–30 years revealed calcium (44% of males and 71% of females) and iron (38% of females) as key nutrient deficiencies, as well as sodium intakes that are well above the adequate intake (AI) level in both sexes [9].

Reports of university students in Australia and the USA, averaged from across all faculties of study, indicate that almost 40% are overweight or obese, and a large proportion do not meet physical activity guidelines (38% in Australia and 54% the USA) [5,10]. While these rates are lower than those among the general adult population in Australia (i.e., 67% are overweight or obese and 55% do not meet physical activity guidelines [11,12]), being overweight or obese in young adulthood is a significant predictor of obesity in later life and has long-lasting effects on physical and mental health [13]. Likewise, physical inactivity in young adulthood may track into adulthood and raises significant concerns for cardiometabolic health [14,15]. Biochemical risk factors have rarely been explored in university students. Among Midwest and Southwest American university students, 2.2–3.9% met criteria for diabetes mellitus [16], and, among Kenyan university students, 1.9% met criteria for metabolic syndrome [17]. In Australia, national data indicate that 0.6% of the young adult population (aged 15–24 years) have diagnosed diabetes [18], but rates of insulin resistance in the general young adult population have not been reported.

While biomedical students are generally considered a “health conscious” group [19], there are no previous Australian reports of their adherence to nutrient and physical activity guidelines, nor their current metabolic health status. Adherence to dietary and physical activity guidelines offers considerable health benefits, mitigates noncommunicable disease risk, and lowers economical costs in the context of both personal and public healthcare [20,21]. To this end, this study aimed to assess adherence to fruit and vegetable, nutritional, and physical activity guidelines in Australian undergraduate biomedical students. Associations between EDNP foods, fruit and vegetable intake, and physical activity levels with body composition and circulating glucose and insulin levels were also assessed.

## 2. Materials and Methods

The study was approved by Bellberry Human Research Ethics Committee (Project approval 2016-02-066-PRE-3) and conducted in accordance with the National Statement on Ethical Conduct in Human Research (Australia).

### 2.1. Study Participants

Participants were recruited from third-year biomedical practical classes from The University of Queensland over 2018–2019. Students who provided written informed consent were provided with a unique, unidentified code (and password for the online diet questionnaire). Inclusion criterion was 19–25 years of age (150 males and 211 females). There were no specific exclusion criteria.

### 2.2. Dietary Intake

The Automated Self-Administered Dietary Assessment Tool (ASA24-Australia-2016) was used to guide participants through a 24 h recall for the previous day [22]. Participants were asked to recall all foods, drinks, and supplements consumed from midnight to midnight. Participants selected an eating occasion from a pre-determined list (e.g., “breakfast” or “snack”) and reported all foods and beverages consumed at that time. Foods and beverages were entered by using specific search terms and selecting items from a returned list. Details of food types, preparation methods, portion sizes, and additions were then queried by the system. Participants were prompted to recall frequently omitted and forgotten foods, and to complete a final review of all items consumed. The nutrition data included in this analysis include those from all foods, drinks, and supplements. Non-genuine entries (e.g., implausible energy intake, duplicate meal entries, insufficient time spent completing the survey) were excluded from the dietary analysis (*n* = 4 males, *n* = 2 females). 

Fruit and vegetable intakes were examined, whereby one serve of fruit was defined as 150 g (including dried fruit, excluding commercial fruit juices) and one serve of vegetables was defined as 75 g (including legumes and beans, excluding potato fries/chips), as per the Australian Dietary Guidelines [23]. These were compared with age- and sex-specific recommendations for the minimum number of serves per day (fruit: ≥2 serves for males and females; vegetables: ≥6 serves for males and ≥5 for females) [23]. Energy-adjusted intake was also calculated as grams of fruit or vegetables consumed per 1000 kJ of total energy intake. Classification of EDNP foods was inferred from the Australian Dietary Guidelines (Guideline 3; [23]) and included a subset of nine food items, including packaged snacks (e.g., potato crisps, muesli bars), confectionery (e.g., ice-cream, chocolate, lollies), baked sweets (e.g., desserts, cakes, muffins), sweetened drinks (e.g., soft drinks, cordial), fatty/processed meats (e.g., sausages, bacon, ham, meat pies), fast food/takeaway (e.g., hot chips, pizza), spreads and sauces (e.g., butter, margarine, jam, cream cheese, mayonnaise, tomato/barbeque sauces, commercial dressings), alcoholic drinks, and miscellaneous (e.g., coffee, tea) [7,24]. Mixed meals with the occasional EDNP ingredient, e.g., pea and ham soup or fried rice with vegetables and bacon, were not regarded as EDNP foods. The percentage of students who consumed any caffeine was reported and, of those, the median amount consumed was calculated. The percentage of students who exceeded national recommendations for alcohol consumption in a single day (i.e., >4 standard drinks or >40 g alcohol) was calculated. The percentage of students who did not report any food items for the following three main meals was also examined: breakfast or brunch (if only coffee was consumed, this was considered a skipped meal), lunch, dinner. Macronutrient and micronutrient intake was compared with age- and sex-specific Nutrient Reference Values for Australia and New Zealand [8]. 

### 2.3. Physical Activity Survey

The Active Australia Survey was used to estimate leisure-time physical activity over the preceding week [25]. Participants self-completed the survey following instruction. Sufficient activity was defined as at least 150 min of activity over at least five sessions per week. Total time was calculated by adding time spent in walking (continuously for at least 10 min), moderate activity, and vigorous activity (weighted by two). To distinguish vigorous versus moderate activity, exercise intensity was estimated for each activity based on a metabolic equivalent (MET) score of 3–6 for moderate or >6 for vigorous, where 1 MET was defined as the resting metabolic rate, equivalent to oxygen uptake of 3.5 mL/kg/h [26]. A score for total sessions was calculated by adding the number of sessions of walking (continuously for at least 10 min), moderate activity, and vigorous activity. The median time spent in total moderate activity (sum of walking continuously for at least 10 min and moderate activity) and in vigorous activity was also reported, according to the survey guide [25]. For all calculations, any reported gardening or yard work was not included, and maximum activity capped at 840 min per activity, as per the survey guide. Incorrectly completed records (e.g., total time spent in walking equating to <10 min per session) were excluded from the dataset.

### 2.4. Anthropometry and Body Composition

Height was measured using a portable stadiometer (to the nearest 0.5 cm). Waist circumference was measured using a measuring tape at the level of the umbilicus (to the nearest 0.1 cm). Body fat (to the nearest 0.1%) and body mass (to the nearest 10^−7^ kg) were measured using air displacement plethysmography (BOD POD GS; COSMED, Artarmon, NSW, Australia). BMI was then calculated using body mass (kg) and height (m) (kg/m^2^). BMI was used to categorise individuals as underweight (<18.5 kg/m^2^), healthy weight (18.5–24.9 kg/m^2^), overweight (25–29.9 kg/m^2^), or obese (≥30 kg/m^2^).

### 2.5. Blood Glucose and Insulin Levels

A subset of students fasted overnight and underwent an oral glucose tolerance test (OGTT) between 09:00 and 12:00 h to assess metabolic health in this group (*n* = 78 males, *n* = 118 females). Blood glucose levels were measured using a glucometer prior to (baseline; fasted), and at one and two hours after, a glucose drink (Carbotest, 75 mg/300 mL). A subset of students (*n* = 28 males, *n* = 29 females) also had fasted plasma insulin levels measured at baseline via ELISA (Mercodia; Sapphire Bioscience, Redfern, NSW, Australia). Homeostatic Model Assessment for Insulin Resistance (HOMA-IR) was calculated as a measure of insulin resistance using the following formula: (fasting blood glucose × fasting plasma insulin)/22.5.

### 2.6. Statistical Analyses

All analyses were performed using GraphPad Prism or SPSS. Exploratory analyses were conducted, separately for males and females, examining data distribution and summary statistics. Continuous data were tested for normality using the D’Agostino and Pearson test and reported as mean ± SD for normally distributed variables and median and interquartile range (IQR) for non-normally distributed variables. Percentages are presented for categorical data. Specific diet and physical activity parameters were tested for multicollinearity and found not to be highly correlated (variance inflation factor; VIF < 2.0). Therefore, multiple regression models were used to explore whether the percentage of energy intake from EDNP foods, percentage food intake from vegetables and fruits, and levels of physical activity predicted body composition and/or metabolic health status (body mass, BMI, waist circumference, percentage body fat, blood glucose levels, plasma insulin levels, HOMA-IR). Models were adjusted for student age, sex, ethnicity, and/or day of reported food and drink intake where *p* < 0.2 for each body composition and/or metabolic health parameter in unadjusted models. 

## 3. Results

Table 1 shows the demographic, metabolic, dietary, and physical activity characteristics in males and females. The average age was 20 years for both sexes (IQR: 19–20 for males and 19–21 for females). The majority of students were Caucasian (54–59%), followed by Asian (27–29%). More than 70% of students were of a healthy weight. Approximately 13% of females were classified as overweight or obese and an equal proportion were classified as underweight. More than 20% of males were classified as overweight or obese, with <4% being classified as underweight. Blood glucose levels were within the normal range for all students, apart from one female student who had a 2 h blood glucose level of 11.7 mmol/L. While fasting insulin levels and HOMA-IR were also mostly normal, seven students (12%) had insulin levels greater than 10 mIU/L (range: 10.4–15.3) and 11 students (19%) had HOMA-IR values greater than 2.0 (range: 2.08–3.6), indicating some degree of insulin resistance (BMI classification of those with insulin levels greater than 10 mIU/L: 43% healthy weight, 28.5% obese, and 28.5% did not consent to recording body mass; BMI classification of those with HOMA-IR values greater than 2.0: 55% healthy weight, 9% overweight, 18% obese, and 18% did not consent to recording body mass). 

Due to differences in practical class scheduling, 56% of males and 50% of females reported on meals and drinks consumed on Sunday, and others on a Tuesday. However, there were no differences in absolute energy intake between Tuesday and Sunday recalls (10,309 versus 10,498 kJ in males and 6776 versus 6744 kJ in females, respectively; Mann–Whitney test). 

Percentage of energy intake from EDNP foods or drinks was over 30% for both sexes. The recommended intake of fruit (≥2 serves per day) was met by <8% of males and <16% of females, with the majority (~70%) consuming less than one serve per day. The recommended intake of vegetables (≥6 serves per day for males and ≥5 serves per day for females) was met by ~12% of males and ~8% of females, with the majority (63%) consuming less than two serves per day. Less than 1% of males and only 3% of females consumed the recommended number of serves for both fruit and vegetables. Caffeine was consumed by ~80% of all male and female students at a median of 69–85 mg/day (predominantly from coffee and tea). Alcohol was consumed by more than 20% of males and 14% of females, of which the average amount consumed per day was almost five-times higher in males (84.6 g or ~8.5 standard drinks versus 18.0 g or 1.8 standard drinks). Among all students, 12% of males and 2% of females exceeded national recommendations for alcohol consumption in a single day (Table 1). Of these, 95% reported dietary intake on a Sunday and hence students were more likely to exceed recommendations on a weekend. 

The most commonly skipped meal was breakfast, with almost 20% of males and 12% of females not consuming any food for breakfast or brunch. A small percentage of students (<5%) skipped lunch or dinner (data not shown).

More than 80% of students achieved sufficient levels of physical activity, with an average of 180 min of moderate activity per week for each sex. For vigorous activity, males participated in 120 min per week and females participated in 60 min per week (Table 1).

Table 2 summarises macronutrient intake relative to current recommendations. Fibre was a key macronutrient deficiency for both sexes, with less than 30% of males and only 24% females meeting AI recommendations. For linoleic acid, less than half of all males and females met AI recommendations. In males specifically, protein intake exceeded the acceptable macronutrient distribution range (AMDR) for almost 60% of students and total fat intake was below the AMDR for 53% of students. For other macronutrients, intake levels matched guidelines for the majority of students. 

Key micronutrient deficiencies for both sexes included vitamin A, calcium, and potassium, with less than half of all students meeting the recommended dietary intake (RDI) (Table 3). Other deficiencies for females included thiamine, vitamin B6, iron, and magnesium. Of particular concern were calcium and iron, with the RDI being met by only 16% and 6% of female students, respectively. Using the estimated average requirement (EAR) as the reference, calcium and iron intakes were met by 23% and 59% of female students, respectively. Zinc was the only nutrient deficiency specific to males, with less than 40% meeting the RDI. For sodium, almost all students (89–95%) exceeded the AI level, and the majority of males (64%) and almost half of all females (46%) exceeded the suggested dietary target (SDT). 

Intake of dietary supplements was reported by 5.5% (7/128) of males and 11.7% (23/196) of females, of which one male and seven females consumed two different types of supplements. The most commonly reported supplement for both sexes was a multiple micronutrient supplement, accounting for 37.5% and 20% of all supplements reported in males and females, respectively. Among females, vitamin C, fish oil, and iron each made up 16–20% of all supplements reported. Other supplements included biotin, vitamin A, vitamin B12, folic acid, horseradish, probiotics and zinc (reported by one to two females only), and creatine monohydrate (reported by one male only).

Multiple regression models were used to explore the association between diet and physical activity with body composition (Table 4) and metabolic outcomes (Table 5). A higher relative intake of fruit was associated with reduced body mass and waist circumference in the unadjusted models (*p* < 0.05) but not when adjusted for sex and/or ethnicity. A higher relative intake of vegetables was associated with higher body mass in both models (unadjusted *p* = 0.091; adjusted *p* < 0.05). Increasing vigorous activity was also associated with higher body mass (*p* < 0.001), BMI (*p* = 0.076), and waist circumference (*p* < 0.05) in the unadjusted models, but not when adjusted for ethnicity, age, and/or sex. Increasing vigorous activity was associated with reduced percentage body fat in both models (unadjusted *p* < 0.001; adjusted *p* = 0.094). Consumption of EDNP foods and moderate physical activity were not associated with any body composition parameter. 

Higher relative fruit intake was associated with higher 1 h blood glucose levels during the oral glucose tolerance test in both models (unadjusted: *p* < 0.01, adjusted *p* < 0.05). Increasing vigorous activity was associated with reduced 1 h blood glucose levels in both models (unadjusted *p* < 0.01; adjusted *p* < 0.05) and reduced 2 h blood glucose levels in the unadjusted model (*p* < 0.05) but not when adjusted for sex. Consumption of EDNP foods, vegetable intake, and moderate physical activity were not associated with any metabolic parameter.

## 4. Results

In a sample of undergraduate biomedical students in Australia, we show that only 1 in 10 met fruit or vegetable recommendations, with less than 1% of males and 3% of females meeting recommendations for both. Furthermore, more than 30% of total energy intake came from EDNP foods. Dietary intake of some nutrients was well below recommendations, including fibre, calcium, and potassium in both sexes, and iron in females. The majority of students, however, achieved sufficient levels of physical activity, with increasing vigorous activity associated with a small reduction in percentage body fat and blood glucose levels. Our data suggest that targeted health promotion messages focusing on fruit and vegetable intake in both sexes, and iron-rich food sources in females, are required to ensure that young adults meet nutrient recommendations.

The proportion of students meeting fruit recommendations in our study (<8% of males and <16% of females) was considerably lower than that reported in the general young adult population (45% of males and 48% of females, 2017–18 National Health Survey, NHS, Australia) [27] and other cross-sectional studies of Australian university students from all faculties of study (51% for both sexes averaged) [5] and first-year medicine (44% for both sexes averaged) [6]. This may be due to differences in data collection methods, whereby other reports queried usual serves per day in whole serves (e.g., “How many serves of fruit do you usually eat each day?”) [5,6,27], which may bias participant reporting by “rounding up” to whole serves. In our study, all 24 h food entries were methodically classified as a fruit item or not, and precise servings per day calculated using weight in grams. This discrepancy has been reported elsewhere, whereby estimates of fruit and vegetable intake based on survey-style questions were higher than those from a 24 h dietary recall [28]. However, in our study, the percentage of students meeting vegetable recommendations (~12% of males and ~8% of females) was higher than the general young adult population (2.5% of males and 5.7% of females, 2017–18 National Health Survey, NHS, Australia) [27] and consistent with other Australian university students (10.5–16%) [5,6]. Nevertheless, overall consumption of fruit and vegetables in this group of students was considerably below recommendations. 

Higher intakes of fibre have been well-documented to reduce the risk of cardiovascular disease and certain types of cancers [29,30,31], and dietary fibre intake specifically in early adulthood has been reported to lower the risk of later-life breast cancer in women [32]. In this study, however, less than 30% of students met fibre recommendations. For both sexes, especially males, this was likely at the expense of excessive protein intake, which in itself is associated with numerous health risks, including kidney and liver dysfunction, and increased risk of cardiovascular disease and some forms of cancer [33]. 

Intake of calcium was also notably low in both sexes, and especially in females, with only 16% and 23% meeting the RDI and EAR, respectively. This is slightly below the national average for young adult females (aged 19–30 years), where only 29% met the EAR [9]. Adequate calcium throughout young adulthood may help to optimise and maintain peak bone mass [34], and numerous studies have reported that adequate calcium intake, particularly via supplementation, is associated with lower blood pressure in young adulthood [35]. In a qualitative study, the perception of young Canadian adults (18–34 years) was that calcium intake is important for children and older adults, but they were uncertain about their own age group [36]. This is consistent with evidence from the USA, where mean daily calcium intake significantly decreased between the ages of 16 and 20 years in more than 60% of males and females [37]. Indeed, national figures estimate that 86% of Australian adults (19–50 years) do not meet the recommended daily serves for dairy and alternatives [38]. 

In our study, only ~30% of males and females met the RDI for potassium, and 64% and 46% of males and females, respectively, exceeded the SDT for sodium. In fact, the average daily intake for sodium was well above the AI level of 460–920 mg (i.e., 2817 mg in males and 1925 mg in females), and only slightly below national data for the general young adult population (3025 mg in males and 2289 mg in females aged 19–30 years) [9]. Sufficient intakes of potassium have been reported to control, and potentially prevent, high blood pressure across the lifespan, especially where sodium intakes are high [39,40,41,42]. Furthermore, in a population of young adults who were mostly normotensive, a higher urinary sodium/potassium ratio was associated with left ventricular hypertrophy [43]. 

Iron deficiency is the most common micronutrient deficiency worldwide, particularly in children and premenopausal women [44]. In the current study, only 6% of females met the RDI for iron. When comparing to the EAR, however, 59% of females met recommendations, which is consistent with the national average of 62% in females aged 19–30 years [9]. Latest data from the World Health Organisation estimate that ~8.5% of Australian women of reproductive age have anaemia [45], with iron deficiency anaemia being a major cause [46]. Interestingly, of the 12% of females that reported intake of one or more supplements, more than half consumed a multivitamin or iron-specific supplement, suggesting some degree of awareness of the importance of iron intake. Increasing consumption of fruits and vegetables is likely to result in reduced protein intake, as well as improving fibre intake, and correcting other micronutrient deficiencies reported in this study. Among females, this should be balanced with higher intakes of iron, particularly heme iron, which has greater bioavailability [47]. 

The consumption of EDNP foods contributed to ~31% of total energy intake in both male and female students. This is only slightly below the national average in young adults (19–30 years), where 36% of energy intake came from discretionary foods [48], and consistent with a recent report in Australian university students from a range of study areas (the majority from Education and Arts), where 32% of energy intake came from EDNP foods [7]. Consumption of EDNP foods was associated with more frequent purchasing of foods and drinks on-campus [7]. Among students enrolled in a nutrition class in Australia [49], consumption of takeaway food was less frequent than that reported in other studies in young adults [50,51], and, in a cohort of Romanian medical students, the majority reported that they consumed fast food either not at all or only once per week [52]. Furthermore, a Spanish study comparing biomedical and non-biomedical university students reported that biomedical students made healthier food choices [19]. Thus, it was somewhat surprising that our population of biomedical students had relatively high consumption of EDNP foods (i.e., consistent with the general young adult population), although takeaway food consumption per se was not examined. While the consumption of EDNP foods was similar between sexes in our study, previous studies have reported that females are more likely to “stress-eat” and consume hyperpalatable comfort foods [53,54,55]. Indeed, the addictive properties of comfort foods can lead to long-lasting changes to eating behaviours [56].

In our study, 12% of males and 2% of females exceeded national recommendations for alcohol consumption in a single day (i.e., more than 4 standard drinks on a single occasion), noting that all but one of these students reported intake for a Sunday. For “usual” number of drinks consumed on a single occasion, 30% of Australian university students from a range of study fields exceeded the single occasion risk, placing them at significant risk of injury each time they drink [5]. Nationally, 67% of males and 55% of females (18–24 years) reported alcohol intake that exceeded the single occasion risk at least once in the past year [57]. 

Dietary habits among university students have been shown to depend on their living arrangement, whereby students living away from home consumed less fruit and vegetables, along with more sugar, alcohol, and fast foods [3,58]. The importance of diet quality goes beyond physical and mental health benefits, with some studies reporting small benefits to academic performance [59]. Among Australian university students specifically, greater intake of fruits and vegetables, and lower intake of EDNP foods, was associated with a higher grade point average (GPA) [60].

With respect to other disease risk factors, we report relatively low rates of overweight and obesity (13–20%) and high rates of physical activity, with more than 80% of students achieving sufficient levels. Nationally, almost half of young Australians aged 18–24 years are overweight or obese [11] and 45% do not meet physical activity guidelines [12]. Similarly, in the previous study of Australian university students from a range of study fields, 40% were overweight or obese, and 38% were insufficiently active [5]. Among Romanian medical students, however, only ~13% were overweight or obese and ~3.2 h per week was spent engaged in physical activity [52]. This latter study is more consistent with our findings, suggesting that biomedical students engage in healthier lifestyle practices than the general young adult and university student population. It is worth noting that, of the 19% of students in our study that had some degree of insulin resistance (HOMA-IR >2), 55% were classified in the healthy weight range, which emphasises the importance of healthy eating and sufficient exercise irrespective of BMI. 

We conducted multiple regressions to determine whether diet and physical activity levels predicted body composition and/or metabolic health. Consumption of EDNP foods, fruits, and vegetables was, by and large, not associated with current body composition and/or metabolic health status. As expected, however, greater levels of vigorous activity were associated with modest reductions in blood glucose levels and percentage body fat, corroborating the importance of high-intensity exercise in the maintenance of metabolic health. Despite few associations, diet and physical activity patterns in the younger years have been reported to track into adulthood [61,62,63]. Furthermore, early-life nutrition has been shown to modulate physical health benefits in the long term, including cardiometabolic disease risk, although the majority of studies have focused on childhood nutrition [64]. 

A key limitation of the current study is that a single 24 h recall may not reflect usual intake. While the majority of students indicated that their intake was of a “usual” amount, they were not queried in relation to usual food types. Underreporting is also a significant issue in dietary studies, with females and people living with overweight or obesity more likely to underreport [65,66]. In this study, however, total energy intake was not affected by BMI in either males or females by linear regression. Given the reported deficiencies in fibre, calcium, and potassium intake, and excessive sodium intake, this may have implications for current cardiovascular and bone health, which should be examined in future studies. Furthermore, some students (26% of all males and 19% of all females) did not consent to measuring body weight and percentage body fat. While the definitive reason is unknown, this could reflect concerns with body image and hence an underreporting of unhealthy BMI categories. 

Universities represent a large setting of young adults—an opportunity for health promotion efforts. Our data suggest that while nutrient deficiencies in young adulthood do not appear to be affecting current body composition and metabolic health parameters, messages focusing on increased fruit, vegetable, and diary intake in both sexes, and increased iron intake among females, are required to optimise overall and lifelong health. 

## Figures and Tables

**Table 1:** Demographic, metabolic, diet, and physical activity characteristics of participants.

	Males *n* = 150	Females *n* = 211
Age [median (range), years]	20.0 (19.0–25.0)	20.0 (19.0–24.0)
*Ethnicity [n, (%)]*		
* *Asian	43 (28.7)	57 (27.0)
* *Asian sub-continental	11 (7.3)	14 (6.6)
* *Caucasian	81 (54.0)	125 (59.2)
* *Multi	3 (2.0)	9 (4.3)
* *Other/Not disclosed	12 (8.0)	6 (2.8)
Body mass ^a^ [median (IQR), kg]	71.3 (65.6–80.0)	59.1 (52.0–66.2)
Height ^b^ [mean (SD), cm]	178 (7.5)	164 (6.9)
*BMI* ^a^ *[median (IQR), kg/m^2^]*	22.8 (20.9–24.7)	21.8 (19.8–23.5)
* *Underweight [*n*, (%)]	4 (3.6)	22 (12.9)
* *Healthy [*n*, (%)]	84 (75.7)	126 (73.7)
* *Overweight [*n*, (%)]	19 (17.1)	17 (9.9)
* *Obese [*n*, (%)]	4 (3.6)	6 (3.5)
Waist circumference ^c^ [median (IQR, *n*), %]	82.0 (77.1–86.9)	74.5 (69.0–80.0)
Body fat ^a^ [median (IQR), %]	15.6 (10.8–21.6)	28.1 (24.3–32.9)
*Blood glucose levels from OGTT* ^d^ *[mean (SD), mmol/L]*		
* *Fasted	4.9 (0.55)	4.7 (0.51)
* *1 h	7.1 (1.3)	8.1 (2.1)
* *2 h	6.0 (1.0)	7.0 (1.4)
Fasted plasma insulin levels ^e^ [median (IQR, *n*), mIU/L]	5.0 (3.5–8.1)	5.5 (4.5–6.7)
HOMA-IR ^e^ [median (IQR)]	1.1 (0.7–1.8)	1.1 (0.8–1.5)
HOMA-IR >2.0 ^e^ [*n*, (%)]	6 (21.4)	5 (17.2)
Energy intake ^f^ [median (IQR), kJ/day]	10,338 (7248–13,245)	6760 (5412–8676)
Energy intake ^g^ [median (IQR), kJ/kg bw/day]	143 (100–184)	113 (89–153)
EDNP foods ^f^ [median (IQR), % total energy intake]	31.3 (15.8–53.9)	31.6 (12.9–48.5)
*Fruit intake* ^f^		
* *Median (IQR), g per 1000 kJ	2.4 (0–14.2)	10.2 (0–28.5)
* *Median (IQR), serves per day	0.13 (0–1.1)	0.46 (0–1.3)
* *<1 serves per day [*n*, (%)]	90 (70.3)	132 (67.4)
* *1 < 2 serves per day [*n*, (%)]	28 (21.9)	33 (16.8)
* *≥2 serves per day [*n*, (%)]	10 (7.8)	31 (15.8)
*Vegetable intake* ^f^		
* *Median (IQR), g per 1000 kJ	5.9 (0–32.1)	14.1 (0–31.9)
* *Median (IQR), serves per day	0.74 (0–4.1)	1.2 (0–3.0)
* *<2 serves per day [*n*, (%)]	81 (63.3)	124 (63.3)
* *2 < 6 (M) or 2 < 5 (F) serves per day [*n*, (%)]	31 (24.2)	56 (28.6)
* *≥6 (M) or ≥5 (F) serves per day [*n*, (%)]	16 (12.5)	16 (8.1)
Meet both fruit and vegetable recommendations ^f^ [*n*, (%)]	1 (0.78)	6 (3.1)
Caffeine consumption ^f^ [*n,* (%)]	100 (78.1)	158 (80.6)
Exceeded single-day alcohol limit > 40 g ^f^ [*n*, (%)]	15 (11.7)	4 (2.0)
Sufficient levels of physical activity achieved ^h^ [*n*, (%)]	114 (85.1)	163 (81.5)
Moderate activity ^h^ [median (IQR), min/week]	180 (100–300)	180 (110–288)
Vigorous activity ^h^ [median (IQR), min/week]	120 (30–300)	60 (0–158)

BMI categories: underweight, <18.5 kg/m^2^; healthy, 18.5–24.9 kg/m^2^; overweight, 25–29.9 kg/m^2^; obese, ≥30 kg/m^2^. Sufficient level of physical activity defined as achieving at least 150 min of activity over at least five sessions per week. BMI, body mass index; EDNP, energy-dense, nutrient-poor; F, female; HOMA-IR, Homeostatic Model Assessment for Insulin Resistance; M, male; OGTT, oral glucose tolerance test. ^a^ *n* = 111 (males), *n* = 171 (females). ^b^ *n* = 149 (males), *n* = 210 (females). ^c^ *n* = 146 (males), *n* = 199 (females). ^d^ *n* = 78 (males), *n* = 118 (females fasting blood glucose), *n* = 115 (females 1 h and 2 h blood glucose). ^e^ *n* = 28 (males), *n* = 29 (females). ^f^ *n* = 128 (males), *n* = 196 (females). ^g^ *n* = 94 (males), *n* = 159 (females). ^h^ *n* = 134 (males), *n* = 200 (females).

**Table 2:** Macronutrient intake and current recommendations.

	Males *n* = 128	Females *n* = 196
Protein intake [median (IQR), g/day] Protein intake [median (IQR), % EI] Meeting RDI [%, (RDI in g/day)] Within AMDR [%, (AMDR recommendation as % EI)] Below AMDR (%) Above AMDR (%)	119 (81.0–173) 27.6 (21.2–33.5) 85.9 (64.0) 33.6 (15–25) 7.8 58.6	72.7 (52.1–95.6) 22.5 (18.6–28.1) 83.2 (46.0) 49.5 (15–25) 10.7 39.8
Carbohydrate intake [median (IQR), g/day] Carbohydrate intake [median (IQR), % EI] Within AMDR [%, (AMDR recommendation as % EI)] Below AMDR (%) Above AMDR (%)	215 (163–312) 51.4 (43.4–60.3) 58.6 (45–65) 29.7 11.7	168 (126–229) 57.2 (48.4–62.0) 65.3 (45–65) 18.9 15.8
Total sugar intake [median (IQR), g/day] Total sugar intake [median (IQR), % EI]	75.5 (51.0–107.5) 16.8 (11.4–23.8)	62.6 (41.2–91.4) 20.2 (14.9–26.5)
Fibre intake [median (IQR), g/day] Meeting AI [%, (AI in g/day)]	23.1 (15.4–32.5) 29.7 (30.0)	18.6 (13.0–24.8) 24.0 (25.0)
Total fat intake [median (IQR), g/day] Total fat intake [median (IQR), % EI] Within AMDR [%, (AMDR recommendation as % EI)] Below AMDR (%) Above AMDR (%)	90.5 (60.8–122.5) 19.8 (15.5–23.6) 43.0 (20–35) 53.1 3.9	64.5 (47.6–89.7) 21.1 (16.7–25.3) 49.5 (20–35) 45.9 4.6
Cholesterol intake [median (IQR), mg/day]	363 (203–651)	230 (136–388)
Saturated fat intake [median (IQR), g/day] Saturated fat intake [median (IQR), % EI] Exceeding % EI [%, (recommendation as % EI)]	30.2 (19.8–45.4) 6.6 (5.0–9.5) 18.0 (<10)	23.6 (15.9–33.1) 7.5 (5.6–9.2) 17.9 (<10)
Monounsaturated fat intake [median (IQR), g/day]	35.3 (21.4–49.3)	24.3 (18.3–36.0)
Polyunsaturated fat intake [median (IQR), g/day]	12.0 (9.0–19.6)	9.3 (6.7–12.8)
Linoleic acid intake, *n*−6 [median (IQR), g/day] Meeting AI [%, (AI in g/day)]	10.1 (7.5–16.2) 38.3 (13.0)	7.8 (5.3–10.8) 47.5 (8.0)
α-linolenic acid intake, *n*−3 [median (IQR), g/day] Meeting AI [%, (AI in g/day)]	1.31 (0.77–2.16) 50.0 (1.30)	0.98 (0.68–1.49) 65.3 (0.80)
Total LC *n*−3 intake, DHA, DPA, EPA [median (IQR), mg/day] Meeting AI [%, (AI in mg/day)]	164 (71.7–343) 50.8 (160)	98.3 (48.5–225) 54.1 (90)

AMDR, acceptable macronutrient distribution range; AI, adequate intake; EI, energy intake; RDI, recommended dietary intake. Recommendations obtained from Nutrient Reference Values for Australia and New Zealand [8].

**Table 3:** Micronutrient intake and current recommendations.

	Males *n* = 128	Females *n* = 196
Vitamin A, _REs_ [median (IQR), µg/day] Meeting RDI [%, (RDI in µg/day)]	596 (305–1181) 33.6 (900)	573 (341–1091) 42.4 (700)
Vitamin B1, _thiamine_ [median (IQR), mg/day] Meeting RDI [%, (RDI in mg/day)]	1.53 (0.98–2.43) 64.1 (1.20)	1.01 (0.62–1.47) 45.9 (1.10)
Vitamin B2, _riboflavin_ [median (IQR), mg/day] Meeting RDI [%, (RDI in mg/day)]	2.11 (1.26–3.07) 73.4 (1.30)	1.34 (0.87–2.10) 62.2 (1.10)
Vitamin B3, _niacin_ [median (IQR), mg/day] Meeting RDI [%, (RDI in mg/day)]	30.0 (19.3–44.4) 80.5 (16.0)	19.0 (11.3–26.0) 65.3 (14.0)
Vitamin B6 [median (IQR), mg/day] Meeting RDI [%, (RDI in mg/day)]	1.71 (1.15–2.75) 70.3 (1.30)	1.20 (0.72–1.65) 39.3 (1.30)
Vitamin B9, _DFEs_ [median (IQR), µg/day] Meeting RDI [%, (RDI in µg/day)]	596 (353–889) 69.5 (400)	392 (236–565) 49.0 (400)
Vitamin B12 [median (IQR), µg/day] Meeting RDI [%, (RDI in µg/day)]	5.16 (3.32–7.58) 85.9 (2.40)	2.91 (1.90–4.88) 61.7 (2.40)
Vitamin C (mg/day) Meeting RDI [%, (RDI in mg/day)]	68.7 (32.1–178) 66.4 (45.0)	56.8 (29.2–130) 57.1 (45.0)
Vitamin E, _α-tocopherol equiv._ [median (IQR), mg/day] Meeting RDI [%, (RDI in mg/day)]	11.4 (7.4–15.5) 58.6 (10.0)	8.1 (5.6–11.7) 60.7 (7.0)
Calcium [median (IQR), mg/day] Meeting RDI [%, (RDI in mg/day)]	754 (500–1,154) 32.0 (1000)	621 (376–814) 16.3 (1000)
Iron [median (IQR), mg/day] Meeting RDI [%, (RDI in mg/day)]	12.8 (9.3–18.6) 79.7 (8.0)	8.8 (6.2–12.2) 6.1 (18.0)
Magnesium [median (IQR), mg/day] Meeting RDI [%, (RDI in mg/day)]	378 (258–530) 44.5 (400)	262 (205–346) 32.7 (310)
Phosphorus [median (IQR), mg/day] Meeting RDI [%, (RDI in mg/day)]	1802 (1205–2349) 82.8 (1000)	1175 (881–1479) 63.3 (1000)
Potassium [median (IQR), mg/day] Meeting RDI [%, (RDI in mg/day)]	3107 (2100–4197) 32.0 (3800)	2233 (1629–3041) 33.2 (2800)
Selenium [median (IQR), µg/day] Meeting RDI [%, (RDI in µg/day)]	110 (75.9–150) 77.3 (70.0)	69.5 (48.3–91.5) 63.3 (60.0)
Sodium [median (IQR), mg/day] Exceeding AI [%, (AI, mg/day)] Exceeding SDT [%, (SDT in mg/day)]	2817 (1733–4426) 95.3 (460–920) 64.1 (2000)	1925 (1237–2713) 89.3 (460–920) 46.4 (2000)
Zinc [median (IQR), mg/day] Meeting RDI [%, (RDI in mg/day)]	12.6 (8.7–19.3) 39.8 (14.0)	8.2 (6.0–10.5) 52.6 (8.0)

AI, adequate intake; DFEs, dietary folate equivalents; RDI, recommended dietary intake; REs, retinol equivalents; SDT, suggested dietary target. Recommendations obtained from Nutrient Reference Values for Australia and New Zealand [8].

**Table 4:** Multiple regression of body composition with diet and physical activity variables. ß-Coefficient (95% CI) reported.

	Model	Body Mass (Grams) ^a^	BMI (kg/m^2^) ^b^	Waist Circumference (cm) ^c^	Body Fat (% Body Mass) ^c^
EDNP food intake (% total kJ)	Unadjusted Adjusted	0.588 (−68.05–69.23) −23.95 (−86.70–38.80)	−0.013 (−0.033–0.007) −0.014 (−0.003–0.006)	0.012 (−0.036–0.059) 0.011 (−0.033–0.054)	−0.032 (−0.084–0.021) 0.028 (−0.071–0.015)
Fruit intake (g/1000 kJ)	Unadjusted Adjusted	**−75.11 (−146.34–−3.88)** * −31.89 (−97.23–33.44)	−0.014 (−0.034–0.007) −0.011 (−0.032–0.010)	**−0.051 (−0.099–−0.003)** * −0.010 (−0.056–0.036)	0.033 (−0.022–0.088) −0.021 (−0.067–0.025)
Vegetable intake (g/1000 kJ)	Unadjusted Adjusted	**54.31 (−8.73–117.35)** ^#^ **59.93 (2.93–116.93)** *	0.010 (−0.008–0.028) 0.011 (−0.007–0.030)	0.012 (−0.033–0.057) 0.026 (−0.016–0.068)	0.016 (−0.032–0.064) −0.003 (−0.043–0.037)
Moderate activity (min/week)	Unadjusted Adjusted	−2.83 (−11.47–5.81) −3.39 (−11.11–4.32)	0.000 (−0.003–0.002) 0.000 (−0.003–0.002)	−0.002 (−0.008–0.004) −0.001 (−0.007–0.004)	−0.003 (−0.010–0.003) −0.003 (−0.009–0.002)
Vigorous activity (min/week)	Unadjusted Adjusted	**20.78 (11.02–30.54)** * 7.76 (−1.56–17.07)	**0.003 (0.000–0.005)** ^#^ 0.002 (−0.001–0.005)	**0.007 (0.000–0.014)** * −0.001 (−0.008–0.006)	**−0.018 (−0.025–−0.010)** * −0.006 (−0.012–0.001) ^#^

ß-Coefficient indicates the unit change for each body composition parameter (dependent variable) per unit increase in each diet and physical activity parameter (independent variables). ^a^ Adjusted for sex and ethnicity. ^b^ Adjusted for sex and age. ^c^ Adjusted for sex. * *p* < 0.05, ^#^ *p* < 0.099 (also designated in bold font). BMI, body mass index; CI, confidence interval; EDNP, energy-dense, nutrient-poor.

**Table 5:** Multiple regression of metabolic outcomes with diet and physical activity variables. ß-Coefficient (95% CI) reported.

	Model	Fasted Blood Glucose (mmol/L) ^a^	1 h Blood Glucose (mmol/L) ^b^	2 h Blood Glucose (mmol/L) ^c^	Fasted Plasma Insulin (mIU/L) ^d^	HOMA-IR ^d^
EDNP food intake (% total kJ)	Unadjusted Adjusted	0.000 (−0.003–0.004) 0.001 (−0.003–0.004)	−0.002 (−0.015–0.010) 0.000 (−0.012–0.013)	0.000 (−0.010–0.009) 0.001 (−0.008–0.009)	−0.008 (−0.045–0.029) −0.012 (−0.049–0.024)	−0.002 (−0.011–0.007) −0.003 (−0.012–0.006)
Fruit intake (g/1000 kJ)	Unadjusted Adjusted	−0.001 (−0.004–0.002) 0.000 (−0.003–0.004)	**0.018 (0.005–0.031)** * 0.015 (0.002–0.028) *	0.003 (−0.006–0.013) −0.003 (−0.012–0.006)	−0.016 (−0.052–0.019) −0.023 (−0.059–0.012)	−0.004 (−0.012–0.005) −0.005 (−0.014–0.003)
Vegetable intake (g/1000 kJ)	Unadjusted Adjusted	0.001 (−0.003–0.005) 0.001 (−0.002–0.005)	0.006 (−0.006–0.019) 0.008 (−0.005–0.020)	0.004 (−0.005–0.014) 0.004 (−0.005–0.013)	0.001 (−0.043–0.044) 0.005 (−0.037–0.048)	0.000 (−0.010–0.011) 0.001 (−0.009–0.012)
Moderate activity (min/week)	Unadjusted Adjusted	0.000 (−0.001–0.000) 0.000 (−0.001–0.000)	0.000 (−0.002–0.001) 0.000 (−0.002–0.001)	0.001 (0.000–0.002) 0.001 (0.000–0.002)	0.001 (−0.003–0.005) 0.001 (−0.003–0.005)	0.000 (−0.001–0.001) 0.000 (−0.001–0.001)
Vigorous activity (min/week)	Unadjusted Adjusted	0.000 (−0.001–0.001) 0.000 (−0.001–0.000)	**−0.003 (−0.005–−0.001)** * −0.002 (−0.004–0.000) *	**−0.001 (−0.003–0.000)** * 0.000 (−0.002–0.001)	0.000 (−0.007–0.006) 0.001 (−0.006–0.007)	0.000 (−0.002–0.002) 0.000 (−0.001–0.002)

ß-Coefficient indicates the unit change for each metabolic parameter (dependent variable) per unit increase in each diet and physical activity parameter (independent variables). ^a^ Adjusted for sex, age, and ethnicity. ^b^ Adjusted for sex and ethnicity. ^c^ Adjusted for sex. ^d^ Adjusted for day of reported intake (Sunday or Tuesday). * *p* < 0.05 (also designated in bold font). CI, confidence interval; EDNP, energy-dense, nutrient-poor; HOMA-IR, Homeostatic Model Assessment for Insulin Resistance.

## Data Availability

The data presented in this study are available on request from the corresponding author.
